# Supplementary material for: Association of Food Intake with Sleep Durations in Adolescents from a Capital City in Northeastern Brazil
Source: Nutrients. 2022 Dec 5;14(23):5180. doi: 10.3390/nu14235180 (PMC9735429; doi:10.3390/nu14235180)
Supplement: Supplementary file 1 [file nutrients-14-05180-s001.zip › nutrients-2022499-supplementary.pdf]

Table S1. Prevalence of consumption of ultra-processed foods with sleep duration in adolescents. São Luís, Maranhão, Brazil, 2016/2017.

**Food consumption**	**Sleep duration**		**p-valor**
	**(≥ 6 hours)** Mean	**(<6 hours)** Mean	
**Ultra-processed**			
Cakes and cookies	7.79	7.90	0.823*
Sweets and candies	5.50	5.11	0.267*
Fast food	4.58	4.92	**<0.001****
Sugar-sweetened beverages	3.92	3.66	0.322*
Salty cookies and potato french fries	3.86	3.51	**<0.000****
Cream cheese/industrialized yogurt	1.40	1.28	0.439*
Margarine	1.15	1.33	**0.025****
Sausage and instant noodles	1.06	1.11	0.543*
Ketchup and mayonnaise	0.51	0.52	**<0.001****
Breakfast cereals and cereal bars	0.53	0.35	**<0.000****

Caption: * Student’s t-test; ** Rank sum test.
